# MetAssign: probabilistic annotation of metabolites from LC–MS data using a Bayesian clustering approach

**DOI:** 10.1093/bioinformatics/btu370

**Published:** 2014-06-09

**Authors:** Rónán Daly, Simon Rogers, Joe Wandy, Andris Jankevics, Karl E. V. Burgess, Rainer Breitling

**Affiliations:** ^1^School of Computing Science, University of Glasgow, Glasgow, ^2^Manchester Institute of Biotechnology, Faculty of Life Sciences, University of Manchester, Manchester and ^3^Institute of Infection, Immunity and Inflammation, University of Glasgow, Glasgow, UK

## Abstract

**Motivation:** The use of liquid chromatography coupled to mass spectrometry has enabled the high-throughput profiling of the metabolite composition of biological samples. However, the large amount of data obtained can be difficult to analyse and often requires computational processing to understand which metabolites are present in a sample. This article looks at the dual problem of annotating peaks in a sample with a metabolite, together with putatively annotating whether a metabolite is present in the sample. The starting point of the approach is a Bayesian clustering of peaks into groups, each corresponding to putative adducts and isotopes of a single metabolite.

**Results:** The Bayesian modelling introduced here combines information from the mass-to-charge ratio, retention time and intensity of each peak, together with a model of the inter-peak dependency structure, to increase the accuracy of peak annotation. The results inherently contain a quantitative estimate of confidence in the peak annotations and allow an accurate trade-off between precision and recall. Extensive validation experiments using authentic chemical standards show that this system is able to produce more accurate putative identifications than other state-of-the-art systems, while at the same time giving a probabilistic measure of confidence in the annotations.

**Availability and implementation**: The software has been implemented as part of the mzMatch metabolomics analysis pipeline, which is available for download at http://mzmatch.sourceforge.net/.

**Contact:** Ronan.Daly@glasgow.ac.uk

**Supplementary information:** Supplementary data are available at *Bioinformatics* online.

## 1 INTRODUCTION

The metabolome, being the entire set of metabolites in a biological system, is a highly informative descriptor of the physiological state of an organism, and understanding the dynamics of the metabolome is essential for a wide range of biomedical applications.

Major advances have been made recently in the development of high-throughput assays to measure the metabolome (Zhou *et al.*, 2012). One of the most popular methods for this purpose is mass spectrometry (MS), coupled to a chromatographic separation, such as liquid chromatography (LC). The output of the LC–MS process is a set of peaks, characterized by their mass per unit charge and their chromatographic retention time. For almost all subsequent analysis to be undertaken, these measured peaks have to be annotated (i.e. matched to the particular metabolites that produced them). Accurate reliable peak annotation and metabolite identification is currently the greatest challenge in high-throughput metabolomics (Dunn *et al.*, 2012). In this article, the terms ‘identification’ and ‘annotation’ are used in the sense specified by the chemical analysis working group of the metabolomics standards initiative, where identification means a positive comparison with an authentic standard using two or more measured quantities (e.g. mass and retention time) and annotation means a positive comparison with compounds using physicochemical properties or spectral databases (Sumner *et al.*, 2007).

Tandem MS (or, more generally, MS*^n^*) and comparison with authentic standards are two common approaches to providing robust metabolite identifications (Sumner *et al.*, 2007). For global untargeted metabolomics, comparison of each detected metabolite with an authentic standard rapidly becomes infeasible (through cost and availability of standards). Fragmentation methods including MS/MS and MS*^n^* are powerful, but rely on libraries of fragmentation patterns of authentic standards (Horai *et al.*, 2010; Ridder *et al.*, 2012; Smith *et al.*, 2005). However, fragment patterns are often similar between isomers of the same compound. Fragmentation prediction algorithms exist (Wolf *et al.*, 2010) but are limited in similarity to standards-derived fragment patterns.

There are three key factors that make peak annotation and metabolite identification difficult. First, the finite mass accuracy of the MS equipment and the large number of potential formulas results in multiple possible mass-matches for each observed peak (Kind and Fiehn, 2006). Second, each metabolite in the sample being measured may produce many peaks, including isotopologues, adducts, molecular fragments and multiply charged ions (Scheltema *et al.*, 2009). These peaks form a dependency structure and exacerbate the problem of overlapping database matches; accounting for them in some manner is needed to avoid an overwhelming number of false annotations. Finally, many observed peaks will be the result of impurities and contaminants (Keller *et al.*, 2008).

A considerable number of computational methods have been developed to address the metabolite annotation challenge (Benton *et al.*, 2008; Brown *et al.*, 2011; Creek *et al.*, 2012; Dunn *et al.*, 2012; Weber and Viant, 2010). One of the main differences among existing algorithms is how they treat derivative peaks and their associated dependency structure. Some methods ignore these relations altogether and match individual peaks against a database (Scheltema *et al.*, 2011). Others attempt to annotate metabolites by first grouping peaks in some manner and then assigning a putative annotation to the groups (e.g. Kuhl *et al.*, 2012; Lee *et al.*, 2013). Additional types of data can also help, e.g. predicted retention times of molecules have recently been used to help in identification (Creek *et al.*, 2011). There has also been much work on the use of multistage MS to produce a ‘fragment tree’ that can be compared against hypothetical fragment trees to contribute to the annotation (Ipsen *et al.*, 2010a; Rojas-Cherto *et al.*, 2012).

As well as methods that attempt to improve accuracy by taking into account inter-peak dependencies, there have also been attempts to incorporate additional information not contained within the spectra to improve annotation. For example, Rogers *et al.* (2009), Silva *et al.* (2014) and Weber and Viant (2010) all investigate the use of metabolic pathway information to improve metabolite annotation.

One aspect that has been largely neglected so far is the inherently uncertain nature of the metabolite annotation task. The level of confidence in putative annotations will vary across metabolites and datasets. For example, the presence of several high-quality peaks of an isotopic series at the same retention time that all unambiguously point towards a particular metabolite should result in a putative annotation that is given higher confidence than an annotation from an isolated noisy peak that could have been produced by any one of a number of metabolites. So far, there has been little effort in developing metabolite annotation methods that provide a quantitative assessment of this uncertainty/confidence in their outputs, with work limited to probabilistic models of isotope intensities (Böcker *et al.*, 2009; Ipsen *et al.*, 2010b), and our previous work (Rogers *et al.*, 2009) (extended by Silva *et al.*, 2014) that relies on knowledge of active metabolic pathways and requires every observed peak to be matched to something in the database.

In this article, we address this shortfall by presenting a method of putative metabolite annotation (MetAssign) that provides probabilistic annotations of individual peaks, as well as a probabilistic estimate of the presence/absence of particular metabolites based on the integration of information from multiple peaks (including isotopes and adducts). The main novelty of this method is in how it explicitly models (through statistical clustering) the dependency structure between peaks in a particular experiment. The method, built within the framework of statistical mixture models, simultaneously groups peaks that are derived from the same metabolite and provides a putative annotation of this metabolite. As well as clustering dependant peaks, the statistical framework of the model provides a natural manner in which to combine the different sources of evidence contained within the spectra (mass per charge, retention time and intensity). MetAssign also opens the door to extensions to other data types, discussed in Section 5.

Finally, we compare MetAssign with two widely used annotation methods across a range of LC–MS datasets from standard chemical mixtures, for which the constituents are known, and demonstrate that the probabilities assigned by MetAssign are well calibrated (the higher the probability, the more likely the annotation is correct) and provide competitive putative annotation performance.

## 2 APPROACH

**Fig. 1.**
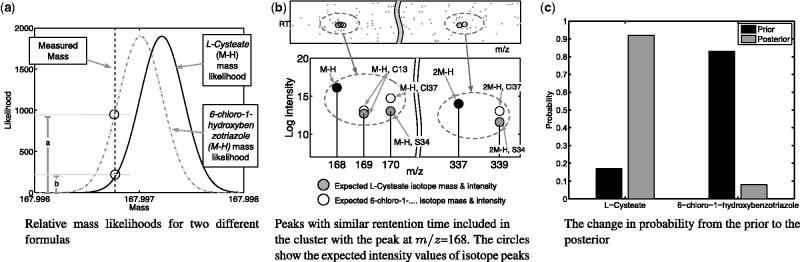
An example of improved peak annotation by MetAssign. The peak at m/z = 167.996769 has two possible database matches, l-Cysteate (which is known to be in the sample) and 6-Chloro-1-hydroxybenzotriazole (which is known not to be in the sample). The prior likelihood of choosing l-Cysteate over 6-Chloro-1-hydroxybenzotriazole is 17 over 83%, and by a nearest match criterion, the later would be selected. However, in the posterior, the ratios change to 92 over 8%, as the MetAssign algorithm detects the confirming presence of the sulphur-34 isotope peaks in the same cluster

Our proposed model adopts a Bayesian statistical approach to peak annotation and metabolite annotation. In particular, we consider annotation as a clustering problem––peaks are clustered into groups, each of which explicitly corresponds to a particular chemical formula. At the peak level, the *prior* probability of a particular annotation is computed via a statistical model based on mass similarity; this is precisely given by the mass likelihood term below. The closer the measured mass to the theoretical mass, the higher the probability. The cluster model described below allows us to convert this *prior* annotation into a *posterior* annotation that takes into account other observed peaks. Posterior probabilities are given by cluster membership probabilities. Figure 1 gives a diagrammatic illustration of this process.

In the following, we describe the statistical model in more detail and show how the output of the cluster model can be interpreted at both the peak annotation and metabolite annotation levels.

### 2.1 Observed data and parameters

Each data replicate consists of *N* mass-chromatographic peaks. Each peak is assumed to have been previously aligned (i.e. matched up) with its corresponding peaks across all *S* replicates. Each peak then consists of the mass-to-charge ratio, *x_n_*; the intensity, *w_n_*; and the retention time, *r_n_*.

As well as data observed from experiments, there are also a library of *m* = 1 … *M* possible metabolite formulas, from which exact masses and predicted isotope profiles can be calculated using a method similar to that described by Snider (2007). Each profile consists of $i=1\ldots I_{ma}$ isotopic indices, where each index consists of the isotopic mass, *y_mai_*, and the isotopic distribution value, β*_mai_* (i.e. the predicted relative intensity based on natural isotope abundances). In addition, possible adduct masses and the corresponding isotope profiles can be calculated using a list of *a* = 1 … *A* possible adduct rules. Each rule is a string such as 2M + 3H, where 2M stands for two copies of the metabolite (dimer) and + 3H stands for an extra 3 Hydrogen atoms (less 3 electrons).

### 2.2 Model description

**Fig. 2.**
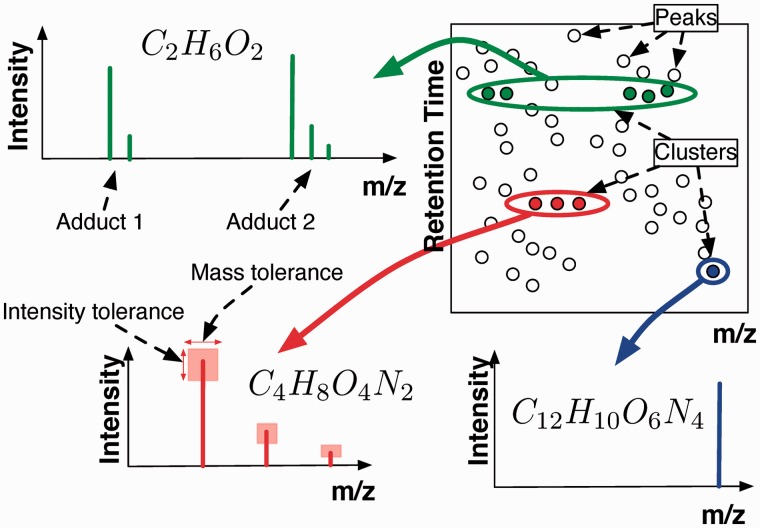
An illustration of the state the model might be in during inference. Three clusters have been highlighted, each grouping around a particular retention time. One of the clusters is made up of two adducts. Each adduct consists of a number of peaks corresponding to the isotopic distribution of the compound

The proposed model simultaneously groups related peaks and assigns molecular formulas to the groups. Inference within the model is performed via a Bayesian Markov Chain Monte Carlo sampling scheme, and the resulting posterior probabilities provide a robust measure of the confidence in particular assignments. An illustration of the state of the model during a hypothetical inference is shown in Figure 2.

At any point in the sampling scheme, we might have *K* clusters, each assigned to a molecular formula and having one or more measured peaks assigned to it. Let the binary indicator variable *z_nk_* = 1, if peak *n* is assigned to cluster *k*, and *z_nk_* = 0 otherwise. Define $c_{k}=\sum_{n}z_{nk}$ to be the total number of peaks assigned to cluster *k*.

Within a cluster we must define the dependencies between peaks, and therefore the exact theoretical peak that a particular measured peak has been assigned to. For example, to use intensity information, it is important to know which isotope peak a particular measured peak is putatively assigned to. In addition, we admit the possibility of multiple adducts in our model and must therefore keep track of which particular adduct a particular measured peak is assigned to. We therefore introduce a second set of indicator variables, *v_nkai_* = 1, if the *n*th peak is assigned to the *i*th isotope position of the *a*th adduct in the *k*th cluster. If there are a total of *I* isotope peaks (note that in general this will depend on the particular chemical formula) and *A* adducts:

$$z_{nk}=\sum_{a=1}^{A}\sum_{i=1}^{I}v_{nkai}.$$

The cluster model takes the form of a mixture model, with a Dirichlet process (DP) prior (e.g. Rasmussen, 2000) to avoid having to specify the number of clusters (metabolites) a priori. The conditional distributions required by the Gibbs sampler to assign peak *n* to a current cluster (*k*) or a new cluster ($k_{*}$) are (note that for brevity we omit conditioning on hyperparameters):

(1) $$P(z_{nk}=1|\ldots )\propto c_{k}p(d_{n}|z_{nk}=1,\ldots )$$

(2) $$P(z_{nk_{*}}=1|\ldots )\propto \alpha p(d_{n}|\ldots )$$

where $d_{n}=(x_{n},w_{n},r_{n})$, *c_k_* is the number of peaks currently assigned to cluster *k*, α is the DP concentration parameter and $p(d_{n}|z_{nk}=1,\ldots )$ is obtained by marginalizing over all low-level assignments possible for the metabolite to which this cluster is linked:

(3) $$p(d_{n}|z_{nk}=1,\ldots )=\frac{1}{A_{\phi_{k}}I_{\phi_{k}}}\sum_{i=1}^{I_{\phi_{k}}}\sum_{a=1}^{A_{\phi_{k}}}p(d_{n}|v_{nkai}=1,\phi_{k},\ldots )$$

where $\phi_{k}=m$ if cluster *k* is linked to formula *m* and we assume uniform priors over the $A_{m}\times I_{m}$ possible adduct and isotope assignments for formula *m*. To compute $p(d_{n}|\ldots )$ for new clusters, we must also marginalize over formulas: see the Supplementary document for information.

Our model assumes that $p(d_{n}|v_{nkai}=1,\phi_{k},\ldots )$ factorizes across the three data types. For the mass term, we assume a Gaussian density on the log of the mass (i.e. mass noise is proportional to *x_n_*):

(4) $$p(x_{n}|v_{nkai}=1,\ldots )=N(\log x_{n}|\log y_{\phi_{k}ai},\zeta^{-1})$$

where $y_{\phi_{k}ai}$ is the theoretical mass of the *i*th isotope peak of the *a*th adduct for the formula assigned to cluster *k*, ζ is the expected precision based on the known accuracy of the specific mass spectrometer used in an experiment, N(b,c) denotes a Gaussian density with mean *b* and variance *c* and N(a|b,c) denotes that density evaluated at *a*.

The intensity term is also Gaussian, but the density depends on the intensities of other peaks currently assigned to this cluster. In particular, we assume that the intensity of adduct *a* in cluster *k*, λ*_ka_*, is drawn from a Gaussian prior $N(\lambda_{0},\kappa_{0}^{-1})$. We set λ_0_ to the mean of observed intensities and κ_0_ to 10^−^^14^, resulting in a fairly flat prior over the region of interest. Individual peak intensities are then assumed to be drawn from a Gaussian conditioned on their adduct-isotope assignment $w_{n}∼N(\beta_{\phi_{k}ai}\lambda_{ka},\kappa^{-1})$, where $\beta_{\phi_{k}ai}$ is the theoretical proportion of total intensity that would be observed as isotope peak *i* and $\kappa =10^{-8}$ is the observation precision. Based on the peaks currently assigned to cluster *k*, we can compute the posterior density over λ*_ka_* (a Gaussian with mean $\lambda_{*}$ and precision $\kappa_{*}$; details in Supplementary document) and then marginalize over λ*_ka_* to obtain the following conditional density that can be used by the sampler:

(5) $$p(w_{n}|v_{nkai}=1,\ldots )=N(w_{n}|\beta_{\phi_{k}ai}\lambda_{*},\kappa^{-1}+\beta_{\phi_{k}ai}^{2}\kappa_{*}^{-1}).$$

For the retention time term, we assume the following generative model: the cluster retention time, *l_k_*, is assumed to be drawn from $N(\mu_{0},\delta_{0}^{-1})$, where $\mu_{0}$ is the mean of the retention times in the data and $\delta_{0}$ is 10^–^^5^. Each peak retention time is assumed to be *l_k_* with additive noise: $r_{n}∼N(l_{k},\gamma^{-1})$, where γ is given as $2.5\times 10^{-1}$. We can analytically compute the posterior density for *l_k_* (a Gaussian with mean $\mu_{*}$ and precision $\delta_{*}$; details in Supplementary document) and marginalize *l_k_* to get:

(6) $$p(r_{n}|v_{nkai}=1,\ldots )=N(r_{n}|\mu_{*},\delta_{*}^{-1}+\gamma^{-1})$$

$p(d_{n}|z_{nk}=1,\ldots )$ is then given by the product of Equations (4–6). The quantity required for a new cluster is computed in a similar manner, but with the posterior parameters replaced by their prior counterparts (for *r_n_* and *w_n_*).

If a peak is assigned to a current cluster, it must then be assigned to a particular adduct–isotope pair within that cluster. The probability of isotope *i* and adduct *a* is:

(7) $$P(v_{nkai}=1|z_{nk}=1,d_{n},\ldots )\propto p(d_{n}|v_{nkai}=1,\ldots )$$

which can be decomposed as above. For a new cluster, we must first assign the cluster to a formula. This is done with:

(8) $$\begin{array}{l} p(\phi_{*}=m|z_{nk_{*}}=1,\ldots )\propto \\ \frac{\pi_{m}}{A_{m}I_{m}}\sum_{i=1}^{I_{m}}\sum_{a=1}^{A_{m}}\;p(d_{n}|v_{nk_{*}ai}=1,\phi_{*}=m,\ldots ), \end{array}$$

where π*_m_* is the probability that a cluster will be assigned to metabolite *m* (π*_m_* = 1/*M* in our experiments), and the assignment to adduct and isotope follows as in the previous case.

The Gibbs sampling algorithm proceeds by starting from a random assignment of peaks into clusters (and particular assignments therein) and then repeatedly re-sampling the assignment for each peak with the various posteriors computed by ignoring the peak being assigned. Note that in practice, the problem is sparse. For each measured peak, the number of theoretical peaks that it could be assigned to [i.e. peaks for which $p(x_{n}|v_{nkai}=1,\ldots )>0$] is small and implementation can be made highly efficient.

This model description demonstrated how MetAssign uses the dependencies present between peaks. To be clustered, peaks must have similar retention times, explainable masses and correct intensity relationships. This distinguishes the method from our previous work (Rogers *et al.*, 2009) and its extensions (Silva *et al.*, 2014) where, for example, peak retention times must be within some tolerance of a theoretical value but are not constrained to be similar to one another.

### 2.3 Annotation probabilities

The output of the Gibbs sampling algorithm is a set of assignments of peaks to a particular mass–adduct–index combination (*mai*)—one for each sample iteration. Based on this, it is straightforward to compute the sample-based approximation to the marginal posterior probability that a measured peak is assigned to any formula/adduct/isotope combination (described here as an annotation). This output may be further adapted to get a better measure of the probability that a measured peak was produced by a chemical compound and was not merely noise. The way in which this idea was implemented in MetAssign was as follows: following each iteration of the Gibbs sampling scheme, we have a series of clusters, each consisting of one or more peaks. For each peak *n*, such that *n* is currently assigned to cluster *k*, an indicator variable $g_{n}\in \{0,1\}$ is calculated as follows:

$$g_{n}=\{\begin{array}{cc} 1 & \text{if}\;g_{i}=1\;\text{ for}\;\text{ all}\;i\;\text{ in}\;k\;\text{ that}\;\text{ have}\\ & \text{a}\;\text{ higher}\;\text{ isotopic}\;\text{ prevalance}\\ 0 & \text{otherwise} \end{array}$$

Thus, a peak is unlikely to be derived from a true metabolite if it is assigned to be an isotopologue peak, but other expected peaks of higher predicted abundance, e.g. the monoisotopic peak, are not detected. In this case, these peaks can be ignored during analysis, by simply using *g_n_* as the peak to metabolite assignments.

On top of the peak sample output, our sampling scheme allows us to produce highly interpretable probabilities of the presence/absence of metabolites with particular chemical formulas. By looking at clusters assigned to a particular formula, we can provide a score for each formula at each sampling step. These can then be averaged over the complete run of samples to provide an overall confidence that this particular formula is present in the data. In the MetAssign program, this behaviour was implemented as follows: at each sample, for each formula *m*, the support for *m* is given as $S_{m}=\sum_{i\in m}g_{i}$, that is, the support is the number of ‘good’ peaks assigned to *m*. We then say that a formula is supported at Level *l* if *S_m_* ≥ *l*. Intuitively, the more peaks are assigned to a formula (e.g. as isotopologues or adducts), the greater the chance that a metabolite with this formula is present.

### 2.4 Using the MetAssign algorithm

Although the description of the statistical model and associated inference algorithm used in MetAssign might seem daunting, the use of the system and interpretation of the output is easy. For each peak in a dataset, a probability that that peak comes from a particular metabolite–adduct–isotope is given. Also, for each compound in a putative database of compounds, the probability that the compound is present in the measured sample is given. The use of probabilities as opposed to definite (yes/no) results might seem to complicate analysis, but in fact this gives a practitioner extremely useful information that allows much greater control in an analysis situation. For example, the user could sum over all adducts and isotopes to find the probability that a peak comes from a particular metabolite, or they could use the maximum a posteriori assignment.

One particular scenario that MetAssign has been designed to perform well in is in large untargeted scans, where a sample contains a large number of metabolites and is being checked against a large database. In situations such as these, common annotation routines end up assigning many compounds to many peaks, and extensive manual intervention is needed (often on a peak-by-peak basis) to resolve inconsistencies that occur. Coupled to this problem is the inverse problem of taking annotations of peaks and deciding whether a compound is present. MetAssign works in this situation by producing groups of peaks that together give more confidence in peak annotations and putative annotations. In situations where there is genuine ambiguity about the annotation of a peak, the probabilities returned will give a measure that can be used directly by the analyst. For example, the user might decide to report only compound matches with probability >80%, or to focus special attention in follow-up experiments on peak groups where two alternative metabolites (formulas) have probabilities close to 50%.

## 3 EXPERIMENTAL METHODS

To examine the behaviour of the annotation algorithm, various experiments were run and the output used to produce summary measures of performance. These experiments included tests of the internal properties of the algorithm (such as robustness and convergence, shown in the Supplementary document) and comparisons of the algorithm against other annotation software packages. The tests were run under different experimental conditions and examined various output properties to produce a robust estimate of how the algorithm would perform in real-life scenarios. The performance of the algorithm was also tested against the performance of two widely used similar metabolite annotation systems, mzMatch and CAMERA (Kuhl *et al.*, 2012; Scheltema *et al.*, 2011).

### 3.1 Comparative evaluation

For the comparative tests, three different standard mixtures of chemical compounds were run as described in the Supplementary Material. Various properties of the output were examined and summarized to produce a quantitative measure of the relative performance of the three algorithms compared. A good algorithm will have the ability to correctly annotate most of the peaks or compounds present in a sample. It will also have the ability to give a low amount of spurious annotations of substances that are not present. There are various statistics that can be used for the comparison: in this article, the measures used will be the recall and precision for analyzing peak annotations, and true-positive rate (TPR, which is the same as recall) and false-positive rate (FPR) for analyzing formula annotations. An algorithm with a high precision and high recall (or, alternatively, high TPR and low FPR) is performing well, though there is normally a trade-off between the two measures. This trade-off can be formalized by combining the measures in some way, e.g. by using the *F*_1_ score. To calculate these values, four other quantities are needed: true positive (TP) (annotated and in the sample), false positive (FP) (annotated and not in the sample), true negative (TN) (not annotated and not in the sample) and false negative (FN) (not annotated but in the sample). The performance descriptors can then be calculated as:

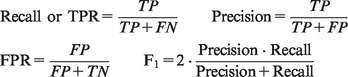


#### 3.1.1 Treatment of output

The output of the mzMatch and CAMERA pipelines consist of possibly multiple compound annotations for each of the peaks in a spectrum. To provide a range of performance values over precision and recall, the distribution of support of a peak for each of the formulas can then be given as 1/|A|, where |A| is the number of annotations on that peak. In terms of metabolite annotation, the output of the mzMatch and CAMERA pipelines does not directly annotate which metabolites are present in a sample. However, given the support distribution on each peak, simply summing the support distribution over all peaks will give a support distribution on each compound. Each compound will then have a vote total between 0 and *N*. Consequently, a threshold can be set, such that all metabolites with votes below the threshold are considered ‘not annotated’ and all with votes equal to and above the threshold are ‘annotated’. A typical value for the threshold would be 1, as this would correspond to at least a single peak uniquely matching a formula.

Because the annotations on MetAssign are probabilistic, there is a natural operating range (from 0 to 1) on which the threshold can be set. This also allows an operating point to be naturally chosen.

#### 3.1.2 Peak annotations

Putative annotation of LC–MS data are normally achieved through the assignment of peaks to one or several compounds. Because, in the analysis described in this article, the compounds in the samples are known, it is possible to create a measure of whether a particular peak is explainable by the sample. If a peak matches a known present compound, this is evidence for a good annotation. If a peak matches a known absent compound, this is evidence against a good annotation. If a peak matches neither a present nor absent compound, this is evidence of noise. From these ideas, the precision and recall can be calculated from a set of annotations of peaks as follows.

For each peak *n*, let *p_n_* be the sum of the support for compounds that are present and let *a_n_* be the sum of the support for compounds that are absent. Discard any values of *p_n_* or *a_n_* that are 0. There then exists a distribution of values that show the relative support for each peak from the annotation database. A threshold can be chosen such that those values above the threshold are positive and those below it are negative. From this, *p_n_* values above the threshold are TP, *a_n_* values above the threshold are FP, *p_n_* values below the threshold are FN and *a_n_* values below the threshold are TN.

In the case of comparing multiple algorithms, the steps above are followed, but instead of discarding all 0 values, only those 0 values for which *p_n_* or *a_n_* are 0 *over all algorithms* are discarded. This ensures the number of items being dealt with (TP + FP + FN + TN) are the same for each algorithm.

### 3.2 Testing conditions

To test the performance of each algorithm at different levels of difficulty of the task, a set of compound databases were used. Each database consisted of the compounds that were known to be present in the sample that was run, plus an extra number of compounds that were not present in the sample. These decoy compounds were chosen to be similar to the compounds in the sample, by finding matches to the compound mass in PubChem within a tolerance of 3 ppm. Database sizes of 100, 600 and 1000 compounds were used. The dataset was pre-filtered at a peak intensity level of 5000. MetAssign was set to output annotations at support levels l = 1 to l = 5; the set of possible adducts used is given in the Supplementary Material.

In addition, the dataset was pre-filtered at peak intensity levels of 0, 5000, 10 000, 15 000 and 20 000, to vary the amount of chemical noise in the data; results for these experiments are given in the Supplementary Material.

For each condition and for each algorithm, the precision and recall for the peaks and the TPR and FPR for the compounds were calculated. This was done over a range of thresholds, to produce precision–recall and Receiver Operating Characteristic (ROC) curves.

## 4 RESULTS

To assess the performance of peak annotations, three measures were used, precision, recall and their harmonic mean, the *F*_1_ score. As in all classification algorithms, there is a trade-off between precision and recall, with good procedures trying to maximize both. With comprehensive results given in the Supplementary Material, Figure 3 shows an example of this trade-off. In these figures, ‘Prior’ means the prior probability of peaks being assigned to metabolites, ‘Posterior’ means the raw posterior probability and ‘MetAssign’ means the posterior probability calculated as described in Section 2.3. As can be seen from these results, MetAssign performs best, with the best precision while recalling the majority of peaks, the target it was designed to achieve. In this circumstance, mzMatch struggles to recall half the peaks, with a precision of 0.5, while CAMERA is extremely selective and annotates only a tiny fraction of the input data, albeit with high precision.

**Fig. 3.**
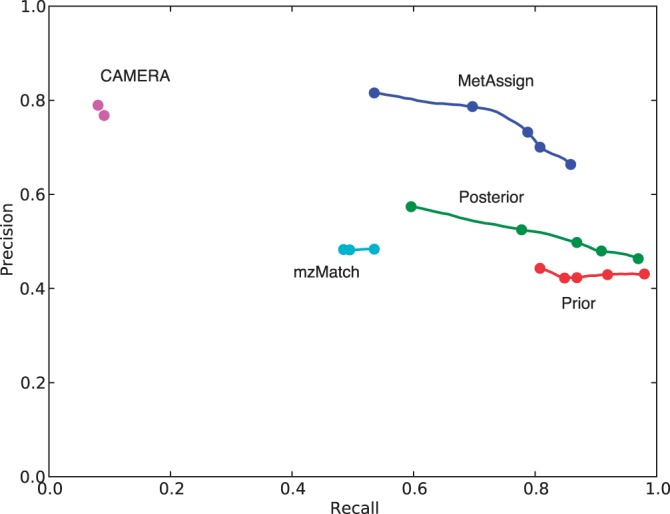
Precision–recall curve for dataset Standard 1, run in negative mode, matched against a database of 1000 decoy compounds. Best possible performance has a precision and recall of 1.0 (top right of figure). The lines run over the useful range of the output (0<threshold≤1), with the marks showing thresholds of 1.0, 0.95, 0.75, 0.5 and 0.0. The lines on the graph show that the behaviour of MetAssign is tuneable to obtain an intended precision/recall value. The behaviour of mzMatch and CAMERA is less tuneable; the default behaviour of these algorithms is given by the rightmost mark on their lines

To see the systematic behaviour of the algorithms, Table 1 shows how the *F*_1_ score changes as the size of the decoy database is varied. For the smallest databases, the prior assignment based on the best match of the mass-to-charge ratio performs best, but as the database grows to more and more realistic sizes, the MetAssign algorithm becomes the best performing method.

**Table 1.** Variation of the *F*_1_ measure over database size and dataset

Dataset	DB size	Prior	Posterior	MetAssign	mzMatch	CAMERA
std1.NEG	100	**0.93**	0.93	0.90	0.68	0.17
	600	0.71	0.74	**0.81**	0.56	0.16
	1000	0.57	0.63	**0.76**	0.49	0.16
std1.POS	100	**0.80**	0.79	0.73	0.53	0.15
	600	0.42	0.48	**0.55**	0.34	0.13
	1000	0.29	0.38	**0.50**	0.27	0.13
std2.NEG	100	**0.90**	0.88	0.83	0.61	0.11
	600	0.64	0.67	**0.70**	0.47	0.11
	1000	0.52	0.58	**0.65**	0.40	0.11
std2.POS	100	**0.85**	0.85	0.83	0.66	0.10
	600	0.35	0.44	**0.52**	0.32	0.09
	1000	0.24	0.34	**0.44**	0.24	0.08
std3.NEG	100	0.66	0.67	**0.69**	0.39	0.15
	600	0.29	0.35	**0.40**	0.21	0.13
	1000	0.22	0.29	**0.34**	0.15	0.13
std3.POS	100	0.51	0.53	**0.54**	0.47	0.18
	600	0.11	0.17	**0.21**	0.11	0.16
	1000	0.06	0.12	**0.17**	0.06	0.15

Bold values indicate best performance.

**Fig. 4.**
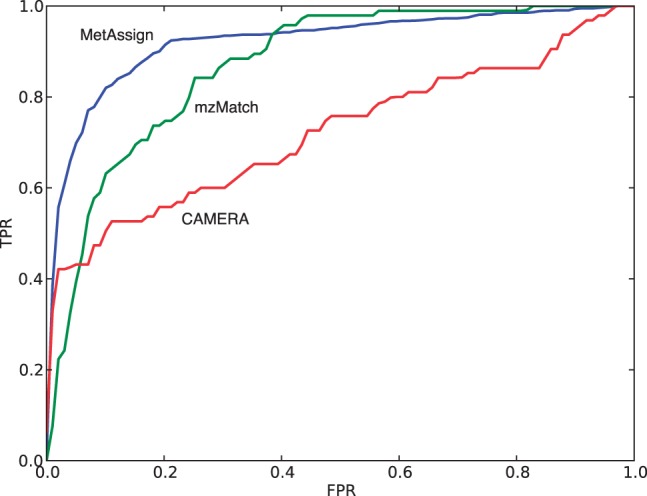
ROC curve for dataset Standard 1, run in positive mode, with an intensity pre-filtering of 5000, matched against a database of 1000 decoy compounds. Best possible performance has a TPR of 1.0 and FPR of 0.0 (top left of figure)

For the metabolite annotation task, the results are presented in terms of the TPR and FPR. An example analysis of this task is shown in the form of a ROC curve in Figure 4. Although the MetAssign output dominates the other methods over most parts of the curve, the operating point will be at a level from 0 to 5% FPR, and it is here that the behaviour is of interest. At this level the number of false positives returned by an algorithm would be limited, while still achieving an acceptable amount of recall of the compounds that are present in the sample. Table 2 shows the performance at an FPR of 5%; as can be seen, MetAssign consistently performs much better than the other two algorithms at annotating metabolites, for all standard mixtures tested and at all sizes of the decoy database.

**Table 2.** Variation of the TPR for compound annotation at a FPR of 0.05, over decoy database size and dataset

Dataset	DB size	MetAssign	mzMatch	CAMERA
std1.NEG	100	**0.78**	0.59	0.41
	600	**0.76**	0.69	0.41
	1000	**0.78**	0.67	0.42
std1.POS	100	**0.74**	0.26	0.45
	600	**0.68**	0.33	0.43
	1000	**0.70**	0.39	0.43
std2.NEG	100	**0.77**	0.48	0.27
	600	**0.77**	0.59	0.26
	1000	**0.79**	0.60	0.26
std2.POS	100	**0.55**	0.45	0.30
	600	**0.49**	0.36	0.31
	1000	**0.49**	0.31	0.29
std3.NEG	100	**0.72**	0.33	0.26
	600	**0.74**	0.33	0.29
	1000	**0.70**	0.38	0.23
std3.POS	100	**0.45**	0.11	0.33
	600	**0.50**	0.11	0.34
	1000	**0.58**	0.11	0.35

Bold values indicate best performance.

## 5 DISCUSSION AND CONCLUSION

We have presented a statistical method for peak annotation and metabolite annotation in large untargeted LC–MS datasets. The novelty in our method lies in the statistical approach to peak annotation that provides a quantitative assessment of the confidence of annotations, as well as probabilities of metabolite presence/absence. Validation studies on real-world experimental datasets showed that MetAssign produced better annotations of peaks and metabolites than two widely used earlier methods, while at the same time providing a measure of confidence in its putative annotations. We believe that these confidence values are useful in subsequent analysis for, e.g. deciding which metabolites warrant further investigation by MS*^n^* or comparison with an authentic standard. It may also be possible to use this system to build a database of metabolites for which putative annotation is generally possible with high confidence.

As alluded to in the introduction, a further benefit of the Bayesian approach is that additional non-conventional forms of information can be easily added to the model. Unlike the MetAssign approach, which seeks to annotate peaks in the presence of derivatives, several recent studies have investigated including metabolic network connectivity into the annotation stage (e.g. Rogers *et al.*, 2009; Silva *et al.*, 2014; Weber and Viant, 2010), and it would be possible to include a connectivity-based prior (such as the one described in Rogers *et al.*, 2009) in the current model. In addition, it has recently been shown that *in silico* retention time prediction can improve annotation, particularly for isomers (Creek *et al.*, 2011). Such information could be incorporated through a metabolite-specific prior retention time distribution, analogous to the metabolite-specific prior isotope intensity distribution. Together, these different approaches provide complementary views of the dataset that can now be integrated in a comprehensive, fully probabilistic pipeline for metabolome data annotation.

Finally, the noise models used throughout MetAssign can almost certainly be improved via better models of the detector itself (Ipsen *et al.*, 2010b) and inclusion in the model of terms relating to contaminants (Keller *et al.*, 2008). The MetAssign algorithm thus provides the basis for the modular development of a general probabilistic framework for the interpretation of LC–MS data in metabolomics.

*Funding*: R.D. was funded by a Netherlands Organisation for Scientific Research Vidi fellowship grant to R.B. J.W. was funded by a PhD studentship from the Scottish Informatics and Computer Science Alliance. This work was supported by the Biotechnology and Biological Sciences Research Council [grant number BB/L018616/1].

*Conflict of Interest*: none declared.

## Supplementary Material

Supplementary Data
