# Supplementary material for: MetAssign: probabilistic annotation of metabolites from LC–MS data using a Bayesian clustering approach
Source: Bioinformatics. 2014 Jun 9;30(19):2764–71. doi: 10.1093/bioinformatics/btu370 (PMC4173012; doi:10.1093/bioinformatics/btu370)
Supplement: Supplementary Data [file supp_30_19_2764__index.html]

MetAssign: Probabilistic annotation of metabolites from LC–MS data using a Bayesian clustering approach — MetAssign: probabilistic annotation of metabolites from LC–MS data using a Bayesian clustering approach — MetAssign: probabilistic annotation of metabolites from LC–MS data using a Bayesian clustering approach — Supplementary Data 

# MetAssign: probabilistic annotation of metabolites from LC–MS data using a Bayesian clustering approach

## Supplementary Data

files

**Files in this Data Supplement:**

- Supplementary Data - pdf file
