# Supplementary material for: MetAssign: probabilistic annotation of metabolites from LC–MS data using a Bayesian clustering approach
Source: Bioinformatics. 2014 Jun 9;30(19):2764–71. doi: 10.1093/bioinformatics/btu370 (PMC4173012; doi:10.1093/bioinformatics/btu370)
Supplement: Supplementary Data [file supp_btu370_supplementary.pdf]

BIOINFORMATICS

Vol. 00 no. 00 2013
Pages 1–6

MetAssign: Probabilistic annotation of metabolites from LC–MS data using a Bayesian clustering approach – Supplementary Material

Rónán Daly [1*], Simon Rogers [1], Joe Wandy [1], Andris Jankevics [2], Karl E. V. Burgess [3] and Rainer Breitling [2]

[1]School of Computing Science, University of Glasgow, UK
[2]Manchester Institute of Biotechnology, Faculty of Life Sciences, University of Manchester, UK
[3]Institute of Infection, Immunity and Inflammation, University of Glasgow, UK

Received on XXXXX; revised on XXXXX; accepted on XXXXX

Associate Editor: XXXXXXX

1 FURTHER MODEL DETAILS

In order to better understand the MetAssign model, a more detailed description is given here. This is similar to the description in Section 2.2 of the article, but is more detailed and also includes a plate diagram as given in Figure 1 and a sampling algorithm as given in Algorithm 1. The algorithm given is a naïve version; a working implementation would recognise that most of the conditional posterior values in p are zero, use log likelihoods, cache frequently used values (such as mass likelihoods) and have a sophisticated $\mathbf{Z}$ and $\mathbf{V}$ structure.

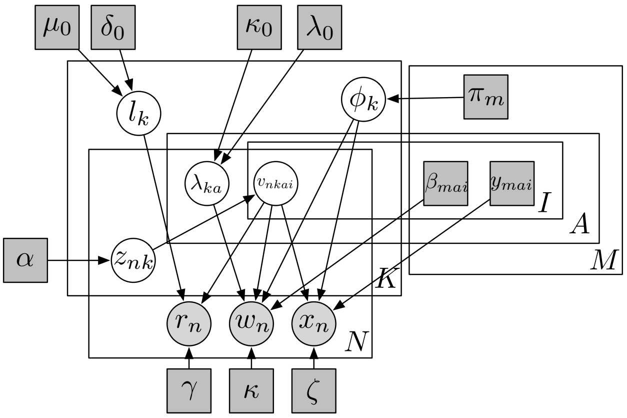

Fig. 1. A plate diagram of the model

The clustering model takes the form of a mixture model with a Dirichlet Process (DP) prior (see e.g. Rasmussen (2000)) to avoid specifying the number of clusters (metabolites) *a priori*. The conditional distributions required by the Gibbs sampler to assign peak n to a current cluster (k) or a new cluster (k_*) are (note that for brevity we omit conditioning on hyper-parameters):

$$P(z_{nk} = 1|\ldots) \propto c_k p(x_n, w_n, r_n|z_{nk} = 1, \ldots) \quad (1)$$

$$P(z_{nk_*} = 1|\ldots) \propto \alpha p(x_n, w_n, r_n|\ldots) \quad (2)$$

where c_k is the number of peaks currently assigned to cluster k, α is the DP concentration parameter and $p(x_n, w_n, r_n|z_{nk} = 1, \ldots)$ is obtained by marginalising over all low-level assignments possible for the metabolite to which this cluster is linked:

$$p(x_n, w_n, r_n|z_{nk} = 1, \ldots) = \frac{1}{A_{\phi_k} I_{\phi_k}} \sum_{i=1}^{I_{\phi_k}} \sum_{a=1}^{A_{\phi_k}} p(x_n, w_n, r_n|v_{nkai} = 1, \phi_k, \ldots) \quad (3)$$

where $\phi_k = m$ if cluster k is linked to formula m and we assume uniform priors over the $A_m \times I_m$ possible adduct and isotope assignments for formula m. For new clusters, the marginalisation is also done over the assignment of the new cluster to a formula (ϕ_*):

$$p(x_n, w_n, r_n|\ldots) = \sum_{m=1}^{M} \frac{P(\phi_* = m)}{A_{\phi_*} I_{\phi_*}} \sum_{i=1}^{I_{\phi_*}} \sum_{a=1}^{A_{\phi_*}} p(x_n, w_n, r_n|v_{nkai} = 1, \phi_* = m, \ldots) \quad (4)$$

In this work, we assume that $\pi_m = P(\phi_* = m) = \frac{1}{M}$.

Our model assumes that $p(x_n, w_n, r_n|v_{nkai} = 1, \phi_k, \ldots)$ factorises across the three data-types. For the mass term, we assume a Gaussian density on the log of the mass (i.e. mass noise is proportional to x_n):

$$p(x_n|v_{nkai} = 1, \ldots) = \mathcal{N}(\log x_n|\log y_{\phi_k ai}, \zeta^{-1}) \quad (5)$$

where $y_{\phi_k ai}$ is the theoretical mass of the ith isotope peak of the ath adduct for the formula assigned to cluster k, ζ is the

*to whom correspondence should be addressed

© Oxford University Press 2013.

Algorithm 1 The sampler used to compute the posterior probability of peak to metabolite assignments. Please note that the data and hyperparameters are implicitly defined.

```
function METASSIGN(S)
    Z, V, φ ← initaliseClustering()
    for s ← 1 ... S do
        order ← shuffle(1 ... N)
        for n ← order do
            METASSIGNPEAK(Z, V, φ, n)
        end for
        samples_s ← V
    end for
    return samples
end function

function METASSIGNPEAK(Z, V, φ, n)
    K ← numberOfClusters(Z)
    for k ← 1 ... K do
        p_k ← CLUSTERPOSTERIOR(Z, V, φ, n, k)
    end for
    p_{k+1} ← NEWCLUSTERPOSTERIOR(n)
    newCluster ← samplePosterior(p)
    setNewCluster(Z, n, newCluster)
    if newCluster = K + 1 then
        for m ← 1 ... M do
            q_m ← METABOLITEPOSTERIOR(n, m)
        end for
        newMetabolite ← samplePosterior(q)
        setNewMetabolite(φ, newCluster, newMetabolite)
    end if
    for a, i ← 1 ... A × I do
        o_ai ← ADDUCTISOTOPEPOSTERIOR(Z, V, n, k, a, i)
    end for
    newA, newI ← samplePosterior(o)
    setAdductIsotope(Z, V, n, newA, newI)
end function

function CLUSTERPOSTERIOR(Z, V, φ, n, k)
    c_k ← clusterSize(Z, k)
    return c_k * p(x_n, w_n, r_n | z_nk = 1, ...)
end function

function NEWCLUSTERPOSTERIOR(n)
    return α * p(x_n, w_n, r_n | ...)
end function

function METABOLITEPOSTERIOR(n, m)
    return p(φ_* = m | z_{nk_*} = 1, ...)
end function

function ADDUCTISOTOPEPOSTERIOR(Z, V, n, k, a, i)
    return P(v_nkai = 1 | z_nk = 1, x_n, w_n, r_n, ...)
end function
```

expected precision based on the known accuracy of the specific mass spectrometer used in an experiment, $\mathcal{N}(b, c)$ denotes a Gaussian density with mean b and variance c and $\mathcal{N}(a|b, c)$ denotes that density evaluated at a.

The intensity term is also Gaussian, but the density depends on the intensities of other peaks currently assigned to this cluster. In particular, we assume that intensity of adduct a in cluster k, λ_{ka} is drawn from a Gaussian prior $\mathcal{N}(\lambda_0, \kappa_0^{-1})$. We set λ_0 to the mean of observed intensities and κ_0 to 10^{-14}, resulting in a fairly flat prior over the region of interest. Individual peak intensities are then assumed to to be drawn from a Gaussian conditioned on their adduct-isotope assignment:

$$w_n \sim \mathcal{N}(\beta_{\phi_k ai}\lambda_{ka}, \kappa^{-1})$$

where $\beta_{\phi_k ai}$ is the theoretical proportion of total intensity that would be observed as isotope peak i and $\kappa = 10^{-8}$ is the observation precision. Based on the peaks currently assigned to cluster k, we can compute the posterior density over λ_{ka}. This is another Gaussian:

$$p(\lambda_{ka}|\ldots) = \mathcal{N}(\lambda_*, \kappa_*^{-1}),$$

where

$$\begin{aligned} \kappa_* &= \kappa_0 + \kappa \sum_{n,a,i} v_{nkai}\beta^2_{\phi_k ai} \\ \lambda_* &= \kappa_*^{-1}\left(\lambda_0\kappa_0 + \kappa \sum_{n,a,i} v_{nkai} w_n \beta_{\phi_k ai}\right) \end{aligned}$$

(note that all summations do not include the peak we are currently sampling assignments for). We can then marginalise over λ_{ka} to obtain the conditional density that can be used by the sampler:

$$p(w_n|v_{nkai} = 1, \ldots) = \mathcal{N}(w_n|\beta_{\phi_k ai}\lambda_*, \kappa^{-1} + \beta^2_{\phi_k ai}\kappa_*^{-1}). \quad (6)$$

For the retention time term, we assume the following generative model: The cluster retention time, l_k is assumed to be drawn from $\mathcal{N}(\mu_0, \delta_0^{-1})$, where μ_0 is the mean of the retention times in the data and δ_0 is 10^{-5}. Each peak retention time is assumed to be l_k with additive noise: $r_n \sim \mathcal{N}(l_k, \gamma^{-1})$, where γ is given as 2.5×10^{-1}. We can analytically compute the posterior density for l_k, which is:

$$p(l_k|\ldots) = \mathcal{N}(\mu_*, \delta_*^{-1}),$$

where

$$\begin{aligned} \delta_* &= \delta_0 + \gamma c_k \\ \mu_* &= \delta_*^{-1}\left(\mu_0\delta_0 + \gamma \sum_n z_{nk} r_n\right). \end{aligned}$$

As for intensity, we can marginalise l_k to get:

$$p(r_n|v_{nkai} = 1, \ldots) = \mathcal{N}(r_n|\mu_*, \delta_*^{-1} + \gamma^{-1}) \quad (7)$$

$p(x_n, w_n, r_n|z_{nk} = 1, \ldots)$ is then given by the product of Equations 5, 6 and 7. The quantity required for a new cluster is computed in a similar manner, but with the posterior parameters replaced by their prior counterparts (for r_n and w_n).

If a peak is assigned to a current cluster, it must then be assigned to a particular adduct–isotope pair within that cluster. The probability of isotope i and adduct a is:

$$P(v_{nkai} = 1 | z_{nk} = 1, x_n, w_n, r_n, \ldots) \propto p(x_n, r_n, w_n | v_{nkai} = 1, \ldots) \quad (8)$$

which can be decomposed as above. For a new cluster, we must also first assign the cluster to a formula. This is done with:

$$p(\phi_* = m | z_{nk_*} = 1, \ldots) \propto \frac{P(\phi_* = m)}{A_m I_m} \sum_{i=1}^{I_m} \sum_{a=1}^{A_m} p(x_n, w_n, r_n | v_{nk_* ai} = 1, \phi_* = m) \quad (9)$$

and the assignment to adduct and isotope follows as in the previous case.

2 DATASETS

The data used for the experiments came from three mixtures of standard metabolites, normally used for identification purposes. These standards (referred to as Standard 1, Standard 2 and Standard 3) contain 104, 96 and 40 metabolites, respectively. Each of these standards was run in triplicate on the system, with interleaving negative and positive ionisation modes. The data from each ionisation mode was gathered to provide the LC–MS profile for that run. The three replicates used were aligned by using mzMatch's Combine algorithm to produce the data used.

The standards were run using ZIC-HILIC chromatography (Merck Sequant, Darmstadt, DE) on an UltiMate 3000 RSLC system (Thermo, Hemel Hempstead, UK), coupled to an Orbitrap Exactive mass spectrometer (Thermo, Hemel Hempstead, UK) in positive and negative ionization mode. The output from each of these runs was transformed to an mzXML file and then to a PeakML file (Scheltema *et al.*, 2011), which was used as input to the algorithms.

The composition of the standards is given in the supplementary information of (Creek *et al.*, 2011, DOI:10.1021/ac2021823)

3 ROBUSTNESS AND CONVERGENCE

Since the MetAssign algorithm is based on a Bayesian framework and is implemented using Monte-Carlo sampling, it is important to check the robustness of inferences to prior parameters. To test prior sensitivity, parameter sweeps were performed, varying the parameters α, γ, γ_m, p_0 and p_1 and no significant changes in posterior values were found.

In order to examine the converge of the Markov chain to its stationary distribution, the MetAssign program was run on each of the standard datasets in positive and negative mode thirty times, each time starting at a random clustering. Each run had a burn in of 600 samples and used 1000 samples for calculating the posterior. The posterior probability for a peak being assigned to each metabolite-adduct-isotope combination was calculated. The standard deviation of these quantities over the thirty runs was calculated. The proportion of these standard deviations under 0.1 are shown in Table 1 and a histogram of these standard deviations for Standard 1 in positive mode is shown in Figure 2. These results show that the vast majority of peak assignments are sampled from the stationary distribution of the Markov chain defined by the sampler.

Table 1. Proportion of assignments of peaks to metabolite–adduct–isotope combinations less than 0.1 over thirty random restarts

Standard 1		Standard 2		Standard 3	
Neg	Pos	Neg	Pos	Neg	Pos
0.987	0.966	0.988	0.977	0.985	0.955

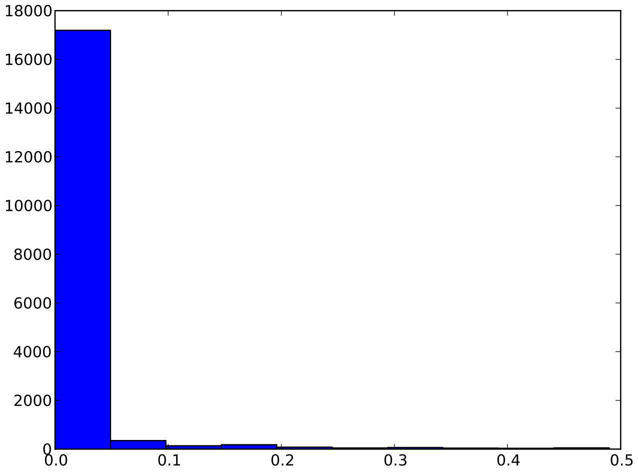

Fig. 2. Standard deviations of assignments of peaks to metabolite–adduct–isotope combinations over thirty random restarts

4 COMPUTATIONAL COMPLEXITY

The runtime of MetAssign depends on a number of factors. The most important of these are the number of peaks N, the size of the database M, the number of adduct types specified A. Strictly speaking, the complexity will be in $O(NMA)$, but because most peaks will match only a very small amount of entries in $M \times A$, the constant in this will be very small. Figure 3 shows the linear relationship between $N \times M \times A$ and runtime. An indicative sample is given as $N = 10334$ peaks, $M = 1079$ compounds and $A = 14$ adduct types, for a runtime of 1259 seconds. Figure 4 shows the relationship between $N \times M \times A$ and memory. Whilst the trend is not as linear as the runtime trend, the relationship is still fairly well determined by this model. The same indicative example as before gives a memory usage of 954 MB. Note that there is a fairly large constant in these figures, dealing with overheads of the executable environment etc.

The execution environment of all the examples given in this paper consisted of an Intel Xeon E5606 running at 2.13 GHz, with 2 GB of available memory.

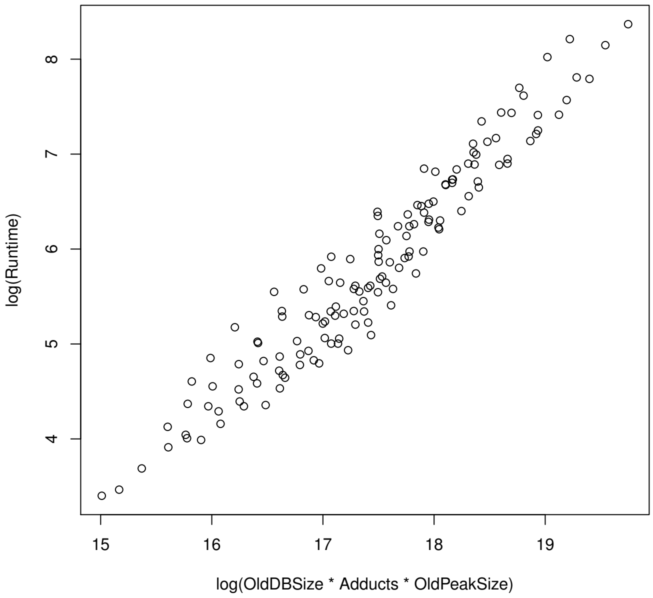

Fig. 3. There is a linear relationship between runtime and $N \times M \times A$, $R^2 = 0.9689$

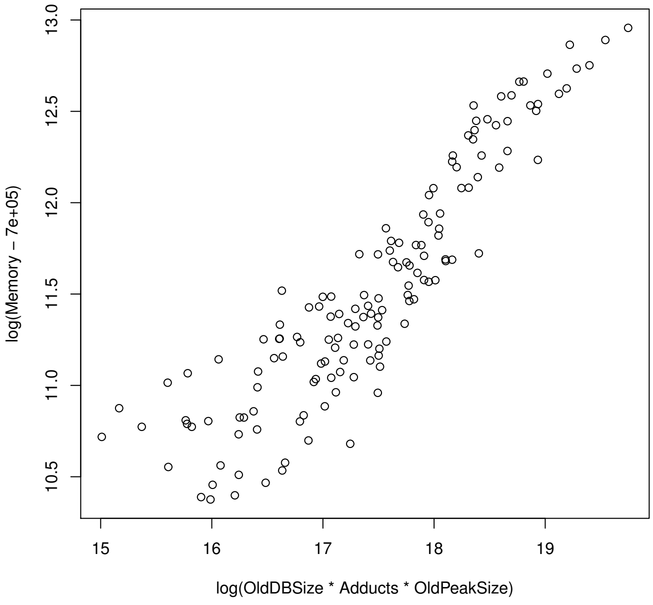

Fig. 4. The relationship between runtime and $N \times M \times A$, $R^2 = 0.8232$

5 RUNNING THE METASSIGN PROGRAM

MetAssign is available as part of the mzMatch suite of tools that operates on data derived from LC-MS experiments. The tools are available at `http://mzmatch.sourceforge.net/`, with a MetAssign tutorial at `http://mzmatch.sourceforge.net/MetAssign.php`. There are many parameters that can modify the behaviour of the program. These will be explained here, though in many cases the default values will work well.

5.1 Running from the command line

Since the mzMatch system is Java-based, a Java Runtime Environment (such as the one provided by Oracle) is needed. Assuming the runtime environment has been set up as explained on the mzMatch website, the following command will run the MetAssign algorithm:

```
JAVA mzmatch.ipeak.sort.MetAssign <parameters>
```

The following is a list of the more important parameters used in MetAssign, with a brief explanation and some guidance to their use. For a full explanation of all parameters, please refer to the mzMatch documentation.

-i <filename>
The PeakML input file

-o <filename>
The PeakML output file containing peak annotations

-sampleOut <filename>
A tab separated output file where compound annotations are stored

-ppm <number>
The PPM accuracy of the measuring mass spectrometer

-filterPPM <number>
Only peak m/z values within filterPPM of theoretical peaks are used

-numDraws <number>
The number of posterior Monte-Carlo draws to collect

-burnIn <number>
The number of posterior Monte-Carlo draws to discard before collection occurs

-databases <comma separated filenames>
A list of databases in mzMatch XML format used for annotation

-adducts <comma separated adduct-formats>
A list of possible adducts that could be formed in the mass spectrometer

-retentionTimeSD <number>
The retention time standard deviation of peaks from their 'true' value

-identificationPeaks <number>
The program will output probabilistic annotations of metabolites supported by at least this many peaks

5.2 Running from R

MetAssign can be used from the mzmatch.R package available from the mzMatch website. From R, the following command can be used:

```
> mzmatch.ipeak.sort.MetAssign(<parameters>)
```

Parameters in this case are passed in the form

```
<parameter name> = <parameter value>
```

An example of this usage is:

```
mzmatch.ipeak.sort.MetAssign(
    i=inputFilename.peakml,
    o=outputFilename.peakml,
    ppm=3
)
```

Detailed instructions on how to run the MetAssign program from R are provided on the mzMatch website at `http://mzmatch.sourceforge.net/MetAssign.php`

5.3 Parameter Usage

When running the MetAssign program, there are a few parameters that should be explicitly set by the user, as these will have a direct influence on the results obtained. These are:

ppm This should be set to the parts-per-million accuracy of the MS equipment. The probability distributions over the theoretical peaks have been defined so that 95% of the probability mass is covered by this value in each direction.

filterPPM This is an optimisation measure to speed up processing by ignoring peaks that are not closer than this value to a theoretical peak. Generally a value between $1.1\times$ppm and $1.5\times$ppm should be appropriate.

numDraws This parameter says how many posterior Monte-Carlo draws to take. For the analysis performed in this paper, 200 samples were taken, which should be sufficient for most uses. For data with more peaks than the test data sets present here or for much larger databases, more samples might be needed, perhaps up to 500.

burnIn This parameters says how many initial Monte-Carlo draws should be discarded before saving posterior samples. For larger datasets, it is recommended to set this to 200.

databases This is a comma separated list of databases that are to be matched against. The format of these databases is XML and is described in the mzMatch documentation.

adducts This is a comma separated list of adduct types that will be used in the generation of theoretical peaks. Whilst an exhaustive list can be provided, it is better to stick with those adducts that are known to be generated, as spurious adducts can generate more false positives.

retentionTimeSD This parameter describes the spread of the retention times of the LC-MS peaks (e.g. isotopic peaks and adduct peaks) that are generated by a single chromatographic peak. This should be set so that the deviation of the retention times of most of these LC-MS peaks from the retention time of the chromatographic peak is less than two times this value. This value can vary widely because of difficulties in detecting accurate retention times from noisy peaks.

identificationPeaks This parameter says to output metabolite annotations that are supported by at least this many peaks assigned to the metabolite.

5.4 Program Output

The output of the MetAssign program consists of two elements: annotations on each of the input peaks of the probability it came from a certain compound and the probability that a certain compound was present in the sample.

5.4.1 Peak Annotation Output The annotated output file (corresponding to the `-o` option) consists of the input file with extra PeakML annotations on each of the peaks. For a description of the PeakML annotation format, please see the mzMatch website.

The particular annotations produced by the MetAssign program consist of STRING annotations on the top-level peaksets that give the probability that a peak came from a certain compound. The three different annotation labels are:

`priorIdentification` corresponding to the prior probability
`probabilityIdentification` corresponding to the basic posterior probability; and
`junkProbabilityIdentification` corresponding to the posterior probability with 'noisy' peaks filtered out.

The format of the strings are as follows:

⟨*annotationString*⟩ ::=⟨*peakIdentification*⟩ '`;`' ⟨*annotationString*⟩
| ⟨*peakIdentification*⟩

⟨*peakIdentification*⟩ ::=⟨*compoundId*⟩ '`,`' ⟨*compoundName*⟩ '`,`' ⟨*adductType*⟩ '`,`' ⟨*isotope*⟩ '`,`' ⟨*probability*⟩
| ⟨*compoundId*⟩ '`,`' ⟨*compoundName*⟩ '`,`' `default` '`,`' ⟨*probability*⟩
| `junk` '`,`' ⟨*probability*⟩

⟨*compoundId*⟩ ::=`string`

⟨*compoundName*⟩ ::=`string`

⟨*adductType*⟩ ::=The adduct type as defined in the main article

⟨*isotope*⟩ ::=⟨*isotopeElement*⟩ ⟨*isotope*⟩ | ⟨*isotopeElement*⟩

⟨*probability*⟩ ::=A floating point number between 0 and 1

⟨*isotopeElement*⟩ ::='`[`' `isotopicNumber elementSymbol` '`]`' `numberOfAtoms`

5.4.2 Compound Identification Output The output for the compound annotation (corresponding to the `-sampleOut` option) consists of a tab-separated file, the rows of which correspond to database compounds and with the following column headers:

`compoundId` The identifier of the compound from the database
`compoundName` The human readable name of the compound
Multiple headers of the form
`p.<number>` Where `<number>`$\geq$ 1: the posterior probability of that compound at support level `<number>`
`p.combined` The mean of the `p.<number>` columns

6 INTENSITY FILTER PEAKSETS

The threshold, against which peaks were filtered, was varied as 0, 5000, 10000, 15000, 20000. This resulted in different peak sets, the sizes of which are shown in Table 2.

7 ADDUCT-TYPES USED IN ANALYSIS

The set of possible adducts used is given in Table 3

Table 2. Number of peaks corresponding to absolute intensity filter level, for each test data set

Filter Threshold	Standard 1		Standard 2		Standard 3	
	Neg	Pos	Neg	Pos	Neg	Pos
0	20527	23859	20354	36577	16466	19546
5000	10241	14527	9792	24916	7287	9165
10000	7194	9583	6505	17592	4848	6672
15000	5778	7477	5200	13370	3956	5407
20000	4883	6166	4342	10334	3383	4539

Table 3. The possible adducts that could be generated. The form of each adduct is explained in the main paper

Positive	M+2H	M+H+NH4	M+ACN+2H	M+2ACN+2H
	M+H	M+NH4	M+Na	M+CH3OH+H
	M+ACN+H	M+ACN+Na	M+2ACN+H	2M+H
	2M+Na	2M+ACN+H		
Negative	M-H2O-H	M-H	M+Na-2H	M+Cl
	M+K-2H	M+FA-H	2M-H	2M+FA-H

8 PEAK INTENSITY FILTERING

In order to examine the results of pre-filtering the data by a peak-intensity thresholding algorithm, each of the data sets was filtered by a set amount and the result of this on the output was examined. For this analysis, the database with 1000 decoy compounds was used. Table 4 shows the results for the F_1 score and Table 5 shows the results for the TPR at 5% FPR. As can be seen, the output can be sensitive to the pre-filtering, as expected. However, independent of the filtering applied, MetAssign outperforms the alternative methods.

9 DATASET PR AND ROC CURVES

This section contains results similar to section 4 of the article, but across all data sets and ionisation modes. Figure 5 gives precision-recall curves whilst Figure 6 gives ROC curves.

REFERENCES

Creek, D. J., Jankevics, A., Breitling, R., Watson, D. G., Barrett, M. P., and Burgess, K. E. V. (2011). Toward global metabolomics analysis with hydrophilic interaction liquid chromatography-mass spectrometry: improved metabolite identification by retention time prediction. *Anal Chem*, **83**(22), 8703–8710.

Rasmussen, C. E. (2000). The infinite Gaussian mixture model. In *Advances in Neural Information Processing Systems 12*, pages 554–560. MIT Press.

Scheltema, R. A., Jankevics, A., Jansen, R. C., Swertz, M. A., and Breitling, R. (2011). PeakML/mzMatch: A file format, Java library, R library, and tool-chain for mass spectrometry data analysis. *Analytical Chemistry*, **83**(7), 2786–2793.

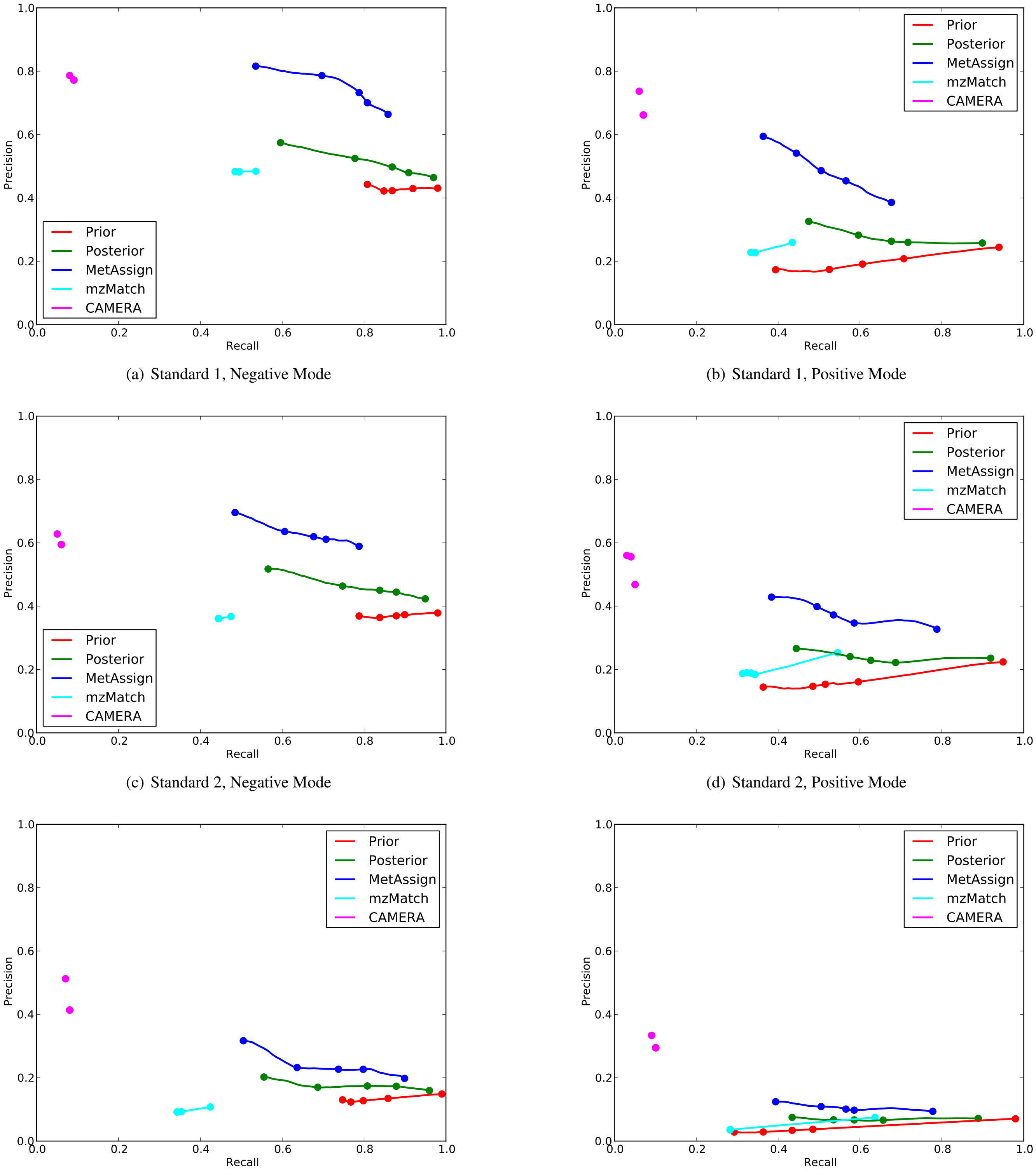

(a) Standard 1, Negative Mode

(b) Standard 1, Positive Mode

(c) Standard 2, Negative Mode

(d) Standard 2, Positive Mode

(e) Standard 3, Negative Mode

(f) Standard 3, Positive Mode

Fig. 5. Precision-Recall curves for all three datasets, run in both modes, matched against a database of 1000 decoy compounds. The lines run over the useful range of the output ($0 < \text{threshold} \leq 1$), with the marks showing thresholds of 1.0, 0.95, 0.75, 0.5 and 0.0. The lines on the graph show that the behaviour of MetAssign is tuneable to obtain an intended precision/recall value. The behaviour of mzMatch and CAMERA is less tuneable; the default behaviour of these algorithms is given by the rightmost mark on their lines.

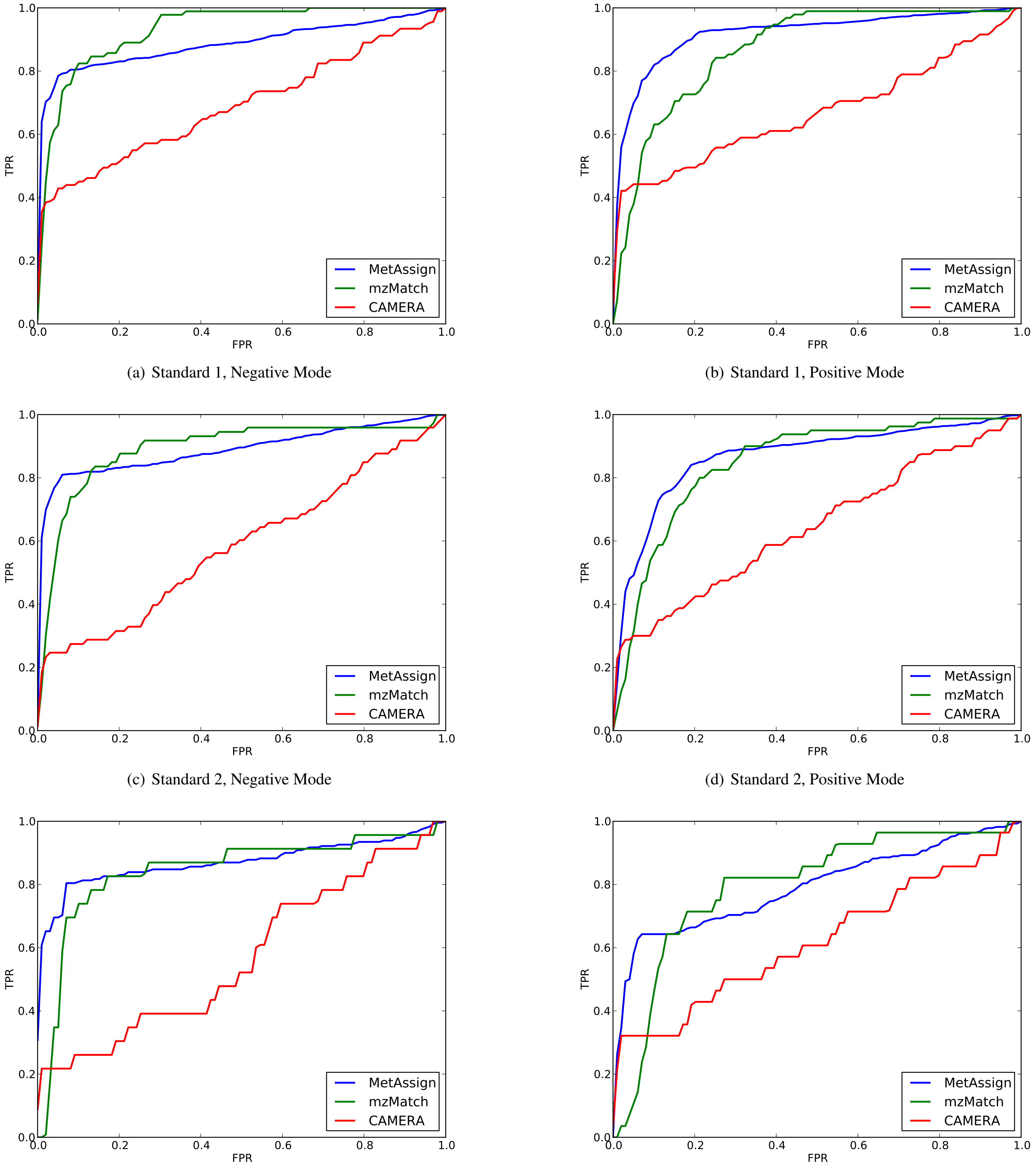

(a) Standard 1, Negative Mode

(b) Standard 1, Positive Mode

(c) Standard 2, Negative Mode

(d) Standard 2, Positive Mode

(e) Standard 3, Negative Mode

(f) Standard 3, Positive Mode

Fig. 6. ROC curves for all three datasets, run in both modes, with an intensity pre-filtering of 5000, matched against a database of 1000 decoy compounds

Table 4. Variation of the F_1 measure depending on intensity filtering threshold and data set

		Prior	Posterior	Posterior Filtered	mzMatch	CAMERA
std1.NEG	0	0.51	0.57	**0.69**	0.44	0.13
	5000	0.57	0.63	**0.76**	0.49	0.16
	10000	0.63	0.68	**0.78**	0.53	0.18
	15000	0.65	0.69	**0.78**	0.55	0.20
	20000	0.67	0.70	**0.75**	0.57	0.21
std1.POS	0	0.28	0.35	**0.45**	0.27	0.09
	5000	0.29	0.38	**0.50**	0.27	0.13
	10000	0.31	0.42	**0.53**	0.30	0.16
	15000	0.34	0.45	**0.54**	0.31	0.16
	20000	0.35	0.46	**0.56**	0.32	0.18
std2.NEG	0	0.47	0.52	**0.61**	0.36	0.09
	5000	0.52	0.59	**0.65**	0.40	0.11
	10000	0.61	0.64	**0.66**	0.45	0.11
	15000	0.65	**0.67**	0.65	0.46	0.13
	20000	0.69	**0.69**	0.64	0.48	0.13
std2.POS	0	0.23	0.30	**0.39**	0.23	0.07
	5000	0.24	0.34	**0.44**	0.24	0.09
	10000	0.26	0.37	**0.47**	0.26	0.11
	15000	0.27	0.39	**0.46**	0.25	0.11
	20000	0.28	0.40	**0.47**	0.25	0.11
std3.NEG	0	0.23	0.29	**0.41**	0.14	0.12
	5000	0.22	0.29	**0.35**	0.15	0.13
	10000	0.22	0.26	**0.31**	0.14	0.18
	15000	0.23	0.26	**0.29**	0.16	0.14
	20000	0.22	0.26	**0.26**	0.16	0.16
std3.POS	0	0.07	0.13	**0.19**	0.07	0.11
	5000	0.06	0.12	**0.17**	0.06	0.15
	10000	0.06	0.12	0.14	0.06	**0.20**
	15000	0.06	0.11	0.13	0.06	**0.21**
	20000	0.07	0.13	0.14	0.06	**0.22**

Table 5. Variation of the TPR for compound annotation at FPR=0.05 depending on intensity filtering threshold and data set

		Posterior Filtered	mzMatch	CAMERA
std1.NEG	0	**0.77**	0.64	0.40
	5000	**0.78**	0.70	0.38
	10000	**0.75**	0.73	0.34
	15000	**0.69**	0.66	0.34
	20000	0.59	**0.66**	0.33
std1.POS	0	**0.67**	0.41	0.44
	5000	**0.70**	0.39	0.44
	10000	**0.74**	0.42	0.44
	15000	**0.68**	0.44	0.39
	20000	**0.68**	0.48	0.39
std2.NEG	0	**0.82**	0.62	0.29
	5000	**0.79**	0.62	0.25
	10000	**0.68**	0.60	0.23
	15000	**0.65**	0.64	0.23
	20000	0.57	**0.67**	0.25
std2.POS	0	**0.44**	0.29	0.34
	5000	**0.49**	0.32	0.29
	10000	**0.54**	0.36	0.28
	15000	**0.56**	0.34	0.25
	20000	**0.56**	0.31	0.23
std3.NEG	0	**0.91**	0.43	0.30
	5000	**0.70**	0.39	0.22
	10000	**0.61**	0.52	0.30
	15000	**0.55**	0.52	0.26
	20000	**0.44**	0.37	0.26
std3.POS	0	**0.49**	0.21	0.32
	5000	**0.58**	0.11	0.32
	10000	**0.53**	0.11	0.36
	15000	**0.50**	0.11	0.29
	20000	**0.48**	0.14	0.36

Note that the entries in this table *can* be directly compared, as the size of the decoy database is constant
